# Answering Hospital Caregivers’ Questions at Any Time: Proof-of-Concept Study of an Artificial Intelligence–Based Chatbot in a French Hospital

**DOI:** 10.2196/39102

**Published:** 2022-10-11

**Authors:** Thomas Daniel, Alix de Chevigny, Adeline Champrigaud, Julie Valette, Marine Sitbon, Meryam Jardin, Delphine Chevalier, Sophie Renet

**Affiliations:** 1 Department of Pharmacy Paris Saint-Joseph Hospital Group Paris France; 2 Innovation and Transformation Department Information Systems Directorate Paris Saint-Joseph Hospital Group Paris France; 3 Learning, Training and Digital Education and Training Research Center University of Paris Nanterre Paris France

**Keywords:** chatbot, artificial intelligence, pharmacy, hospital, health care, drugs, medication, information quality, health information, caregiver, healthcare staff, digital health tool, COVID-19, information technology

## Abstract

**Background:**

Access to accurate information in health care is a key point for caregivers to avoid medication errors, especially with the reorganization of staff and drug circuits during health crises such as the COVID‑19 pandemic. It is, therefore, the role of the hospital pharmacy to answer caregivers’ questions. Some may require the expertise of a pharmacist, some should be answered by pharmacy technicians, but others are simple and redundant, and automated responses may be provided.

**Objective:**

We aimed at developing and implementing a chatbot to answer questions from hospital caregivers about drugs and pharmacy organization 24 hours a day and to evaluate this tool.

**Methods:**

The ADDIE (Analysis, Design, Development, Implementation, and Evaluation) model was used by a multiprofessional team composed of 3 hospital pharmacists, 2 members of the Innovation and Transformation Department, and the IT service provider. Based on an analysis of the caregivers’ needs about drugs and pharmacy organization, we designed and developed a chatbot. The tool was then evaluated before its implementation into the hospital intranet. Its relevance and conversations with testers were monitored via the IT provider’s back office.

**Results:**

Needs analysis with 5 hospital pharmacists and 33 caregivers from 5 health services allowed us to identify 7 themes about drugs and pharmacy organization (such as opening hours and specific prescriptions). After a year of chatbot design and development, the test version obtained good evaluation scores: its speed was rated 8.2 out of 10, usability 8.1 out of 10, and appearance 7.5 out of 10. Testers were generally satisfied (70%) and were hoping for the content to be enhanced.

**Conclusions:**

The chatbot seems to be a relevant tool for hospital caregivers, helping them obtain reliable and verified information they need on drugs and pharmacy organization. In the context of significant mobility of nursing staff during the health crisis due to the COVID-19 pandemic, the chatbot could be a suitable tool for transmitting relevant information related to drug circuits or specific procedures. To our knowledge, this is the first time that such a tool has been designed for caregivers. Its development further continued by means of tests conducted with other users such as pharmacy technicians and via the integration of additional data before the implementation on the 2 hospital sites.

## Introduction

### Background

The hospital is a particularly complex environment that has to maintain continuous health care activities 7 days a week. A high staff rotation is essential to achieve permanent care, while ensuring proper clinical and pharmaceutical knowledge among staff members. This was even truer during the COVID-19 crisis, when many caregivers had to reorganize themselves and adapt to take care of patients. Moreover, the growing number of marketed drugs and their specificities (ie, drugs’ availability, supply chain, dosage, compatibilities, stability, and administration techniques) has led to an increasingly large amount of available information that must be processed, far beyond practical use and knowledge for most caregivers and patients [1]. Besides, each hospital may have specificities in terms of processes, organization, and medication system. When a question about a drug arises, the health care staff, including nurses, needs to be able to find a full, reliable, and accurate answer to ensure proper and safe care. This research often has to be carried out in a short time in addition to the already existing workload, which further complicates the process [2]. Moreover, accurate information may not be easily accessible, especially during night shifts when there are fewer people available in the health care unit. Poorly documented websites might then end up being the last option to obtain information. This was all the more critical during the COVID-19 crisis when medical misinformation has abounded in numerous websites and forums, particularly about vaccines [3]. In such cases, the lack of access to accurate information can lead to medication errors [4,5]. Especially in hospital settings, any of these errors can have serious clinical consequences for patients.

The hospital pharmacy is then the main intermediary providing pharmaceutical advice. Nevertheless, telephone calls to the pharmacy can lead to a growing number of task interruptions for pharmacists, especially if these questions become repetitive, as a consequence of the high staff rotation, which is particularly frequent among nurses. Answers to these questions can also vary among pharmacists, depending on various factors such as the pharmacist’s experience (senior, intern), his/her specialty, and the sources used for reference. Thus, there is a need for hospital pharmacists to find a new way to centralize, standardize, and internally verify the scientific validity of all answers to drug-related questions asked by caregivers. A computerized tool that would generate automated answers to caregivers, using the potential of artificial intelligence, could solve these issues.

Indeed, virtual assistants, also called chatbots, are already used in the field of human health. It is a very easy-to-use tool that users can access through SMS text messages, on smartphones, on computers, or on other connected devices (it needs an internet connection to function). It looks like a classic conversation window, and both the user and the chatbot can have a discussion in human language. The user asks his/her question directly in the dedicated field or clicks on specific buttons. Chatbots have been shown to be useful and effective to provide information and advice to patients with chronic conditions [6-8] as well as promoting a healthy lifestyle [9]. They are also used for oncological applications, in diagnosis and symptom screening, patient or treatment monitoring, as well as mental health counseling or emotional support [10-13]. However, there is currently no chatbot dedicated to caregivers’ questions about drugs and pharmacy organization. We thought that a chatbot with a pharmaceutically validated database, implemented into the hospital intranet, could provide caregivers accessible and fact-checked answers in their everyday practice.

### Objectives

We aimed at developing and implementing a chatbot, focusing on drugs and pharmacy organization to answer questions from hospital caregivers 24 hours a day and then evaluated it.

## Methods

### Setting

The proof-of-concept study took place in a large, private, nonprofit French hospital (comprising 592 beds) between 2021 and 2022 (1 year). The project was conducted by a multidisciplinary team composed of 3 hospital pharmacists, 2 members of the Innovation and Transformation Department, and an IT service provider.

### Chatbot Design

#### Instructional Design

We used the ADDIE (Analysis, Design, Development, Implementation, and Evaluation) model [14] to set up the chatbot tool in the hospital—this is an instructional systems design model divided into 5 successive and iterative phases.

#### Analysis

During 1 month in February 2021, we asked hospital caregivers from 5 departments—especially nurses and pharmacists—what the most frequently asked questions about drugs and pharmacy organization were in their daily practice. We then synthetized the results to integrate appropriate items into the chatbot.

#### Design and Development

We designed and introduced relevant topics into the chatbot in accordance with the previous needs analysis. To provide reliable and clear answers, different sources of information were cross-checked. The following are various pharmaceutical professional tools at our disposal in France: pharmaceutical references derived from the summaries of product characteristics; local recommendations of the Commission for Medicinal Products and Sterile Medical Devices of the establishment; publications of health authorities such as the Observatories of Medicines, Medical Devices and Therapeutic Innovations, the French National Agency for Drugs and Medicinal Products Safety, and the French National Authority for Health; and reference websites such as Stabilis. Each working document was reviewed and scientifically verified by a hospital pharmacist in the relevant field and then implemented into the chatbot. For drug names, we integrated both international nonproprietary names and brand names in the chatbot, so the tool understood both formulations.

We then developed the chatbot itself, using the node.js runtime environment and typescript language, through regular exchanges with the IT service provider. In order for the chatbot to understand human language and be able to answer, we also used Watson’s application programming interface (IBM Corp). Thus, the chatbot used natural language processing and machine learning to understand the user and learn from their previous interactions. It also used an auto-completion engine developed with the IT provider to help users write the drugs’ names. Hence, when the user entered the first letters of a drug’s name, a list of matching proposals was displayed. Moreover, we integrated a fuzzy matching feature to avoid spelling differences and mistakes.

#### Implementation and Evaluation

We implemented the chatbot in the hospital intranet of 4 tester departments to evaluate it. Hence, the beta test was conducted between January and February 2022 before implementing the tool in all hospital care units. It consisted of 20-minute trial sessions organized within the hospital care services, at the end of which, testers (head nurses, nurses, pharmacy assistants, pharmacy students, and interns who agreed to participate) were asked to complete a questionnaire about themselves and about their experience with the chatbot. They had to rate the chatbot’s speed, usability, and appearance, and they were asked to assess their satisfaction with it on a 5-point Likert scale (from “very unsatisfied” to “very satisfied”). They were also asked what missing topics they wanted to include in the chatbot to improve the tool. In addition, we also measured the performance of the tool from the IT provider’s back office. At the end of each interaction with the chatbot, users were invited to provide positive or negative feedback (thumb up or down). The relevance of the chatbot was defined as the number of positive feedbacks compared to the number of total expressed feedbacks, and we also counted the number of nonexpressed feedbacks.

### Ethics Approval

No ethics approval was necessary for this study, given that the chatbot does not fall within the definition of a medical device within the meaning of European Union regulation 2017/745, and that French legislation (public health code) does not require ethics approval for this kind of work.

## Results

### Needs Analysis

The first step, which consisted of collecting and analyzing the needs of future users, was conducted in the hospital in February 2021. In total, 50 questions asked by 33 nurses, 3 head nurses, and assistant nurses were collected and classified into 7 categories. Five different health care units were included: Oncology Outpatient Service, Oncology Inpatient Service, Weekday Inpatient Oncology Service (WIOS), Pneumo-Oncology Service, and Neonatology Service. We also took into consideration the suggestions of 5 hospitals pharmacists concerning the drug circuits and anticancer drugs. Injectable drug compatibility, stability, and administration methods were the main subjects of these questions. Other topics included therapeutic equivalences and anticancer drugs, as shown in Table 1.

**Table 1:** Topics of the caregivers’ questions during the needs analysis.

	Oncology Outpatient Service	Oncology Inpatient Service	Weekday Inpatient Oncology Service	Pneumo-Oncology Service	Neonatology Service	Total, n (%)
Administration, n	—^a^	3	7	3	—	13 (26)
Availability, n	—	1	—	1	—	2 (4)
Anticancer drugs, n	4	—	—	—	—	4 (8)
Compatibility and stability, n	3	4	1	5	1	14 (28)
Cutting or crushing tablets, n	—	2	—	3	—	5 (10)
Miscellaneous, n	5	1	—	2	2	10 (20)
Therapeutic equivalences, n	—	1	1	—	—	2 (4)
Total, n (%)	12 (24)	12 (24)	9 (18)	14 (18)	3 (6)	50 (100)

^a^Not determined.

### Design and Development

**Figure 1:**
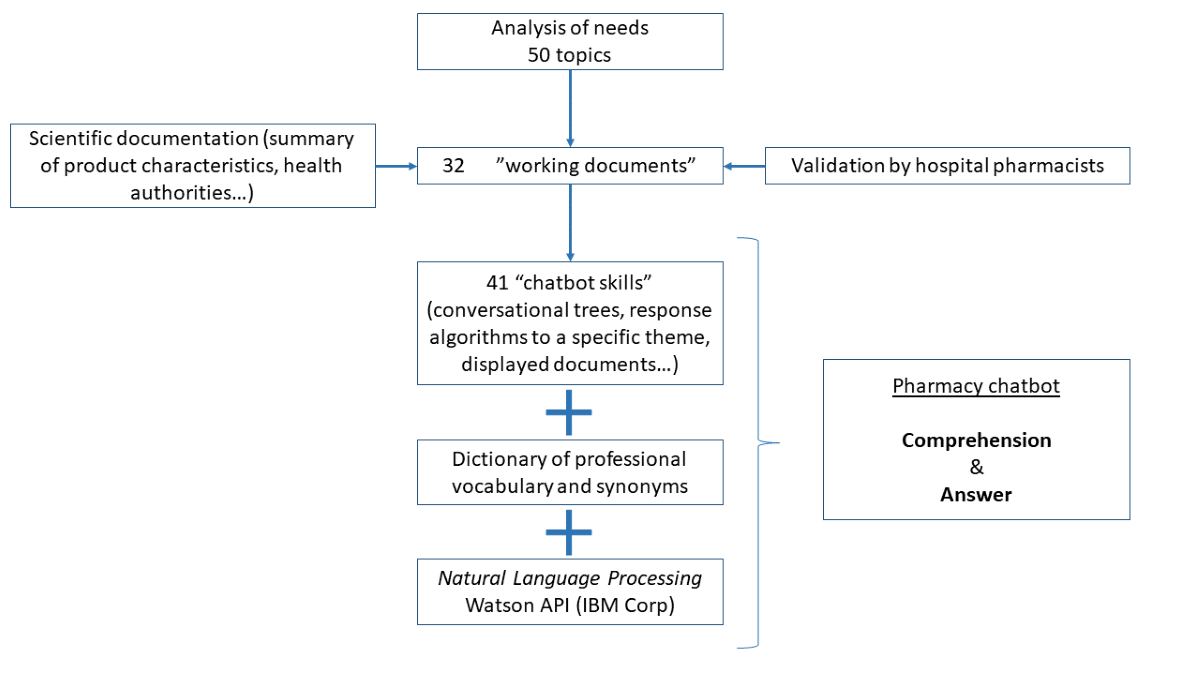
Pharmacy chatbot design scheme. Construction of the chatbot was based on the analysis of the needs of caregivers to integrate relevant data. To understand the questions asked, a natural language processing application programming interface (Watson API, IBM Corp) and a homemade dictionary were also included. API: application programming interface.

The 50 topics indicated during the needs analysis led to the writing of 32 working documents, gathered by item. From the working documents, we created 41 skills into the chatbot, which formed the database with the corresponding resource documents. For each skill, we also built a conversational tree, which constituted a decisional algorithm to determine how the chatbot might interact with the user to provide the requested response (Figure 1). For example, depending on the type of question asked, the chatbot might ask the user for further information, answer directly, or provide a web link to find the correct answer on a reliable website. An example of how the chatbot handles a request concerning the availability of medication is detailed in Multimedia Appendix 1. The chatbot was designed to function in French. We did not provide the tool a name, as it was intended to be integrated later into a global chatbot (meta-bot); we simply called it “Pharmacy chatbot.”

**Figure 2:**
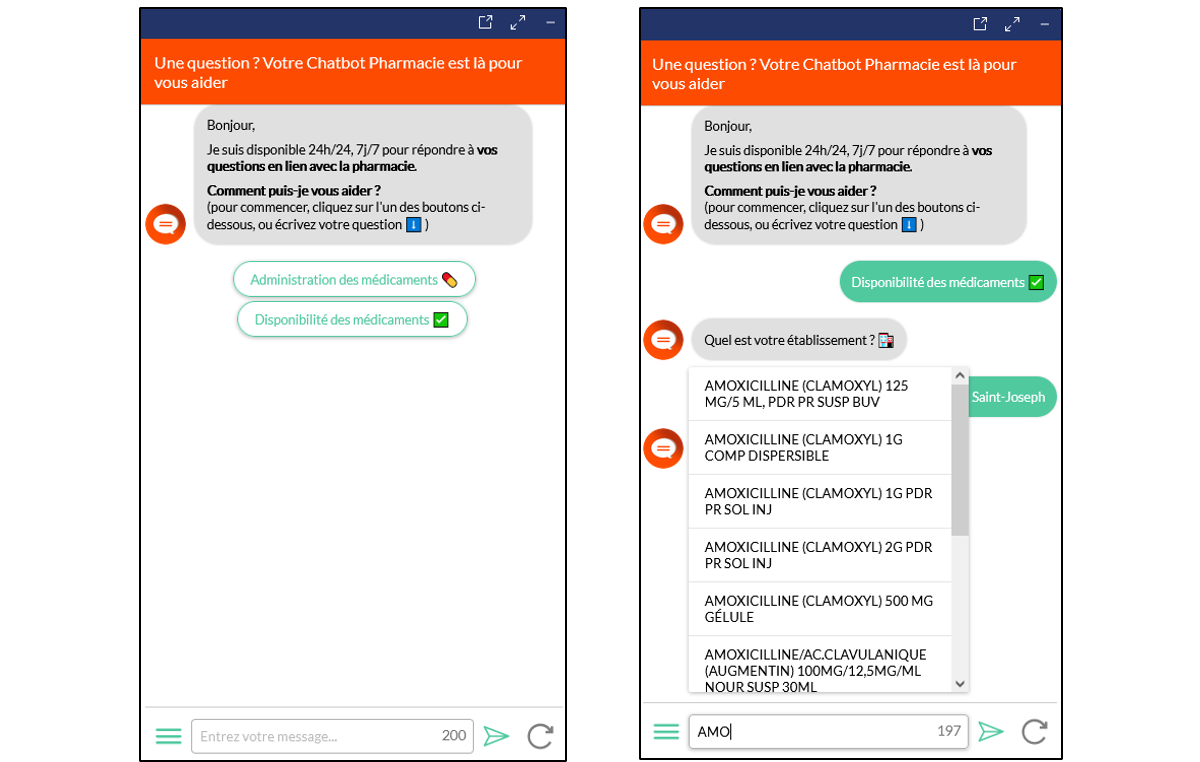
Screenshots of the pharmacy chatbot interface. Left: the opening dialogue is designed to guide the users on frequently discussed topics. Right: the auto completion system helps users write drug names correctly.

We also included in the chatbot an initial dialogue to guide the users more quickly on frequently asked topics and used technical solutions called “fuzzy matching” and “auto completion” to help users formulate their requests and make them understandable by the chatbot (Figure 2).

### Implementation and Evaluation

**Figure 3:**
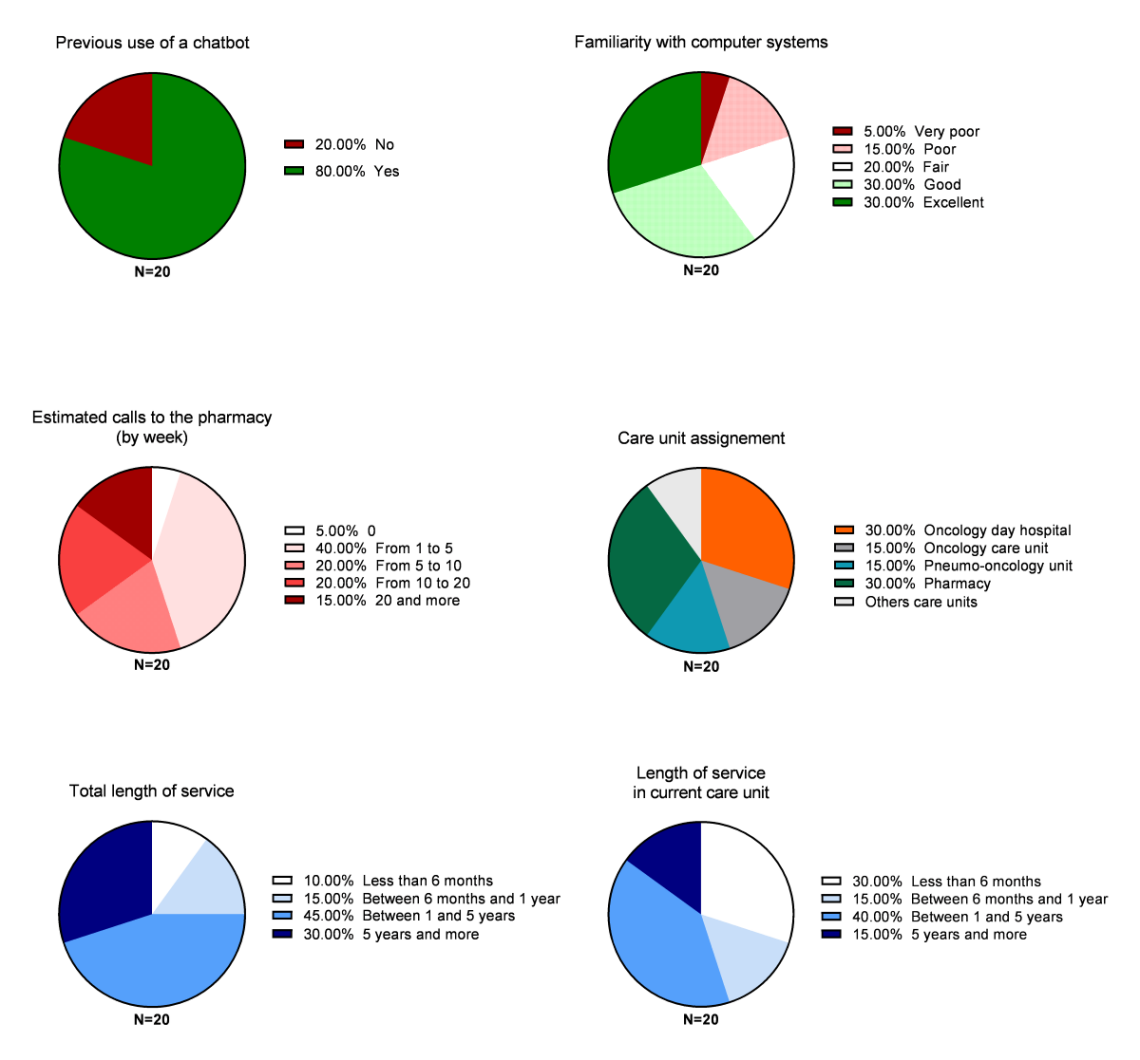
Descriptive analysis of the population of beta testers. Testers were invited to respond a questionnaire with their attitudes toward the previous use of a chatbot, their estimated familiarity with the computer tool on 5-point Likert scale, and the weekly number of calls to the hospital pharmacy. Testers were also invited to indicate their care unit and their experience.

The beta test of the chatbot tool was conducted between January and February 2022. In total, 20 caregivers from 4 different services (Oncology Outpatient Service, WIOS, Pneumo-Oncology Service, and pharmacy department) attempted the proof-of-concept version of the chatbot during test sessions. A total of 14 nurses and head nurses participated, as well as 6 members of the pharmacy staff. The beta test led to 214 conversations, and testers were invited to complete a satisfaction questionnaire, the results of which are presented in Figure 3.

**Figure 4:**
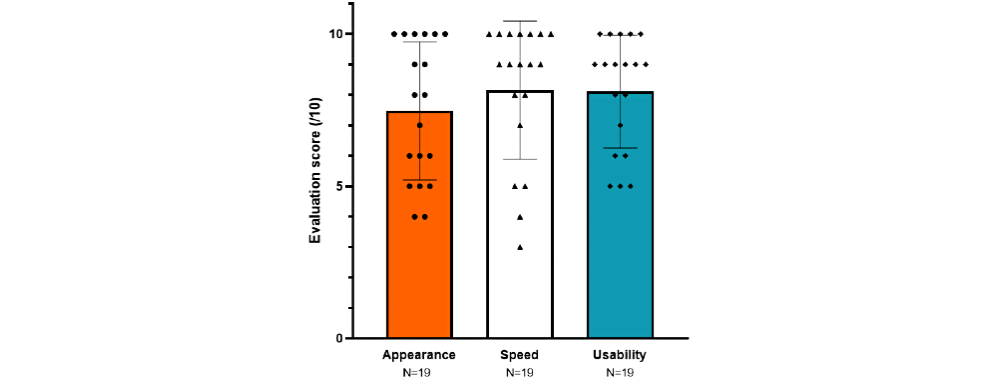
Evaluation scores of the chatbot appearance, speed, and usability after the beta test. Each point matches with an individual value, and vertical lines represent the mean and SD values.

Overall, 8 of 20 (40%) testers used to call the pharmacy 1 to 5 times a week, and 11 (55%) used to do it more. Only one person, an experienced head nurse, said that she never had to call the pharmacy. The chatbot’s speed was rated 8.2 out of 10 (range 3-10), its ergonomics was rated 8.1 (range 5-10), and its appearance was rated 7.5 (range 4-10) as shown in Figure 4. One person did not rate the chatbot. Overall, 14 of 20 (70%) users were satisfied or very satisfied with the tool. In the back office, the estimated relevance (ie, conversations with positive feedback) was 76% for of interactions that were not aborted (146/214, 68% of conversations).

**Figure 5:**
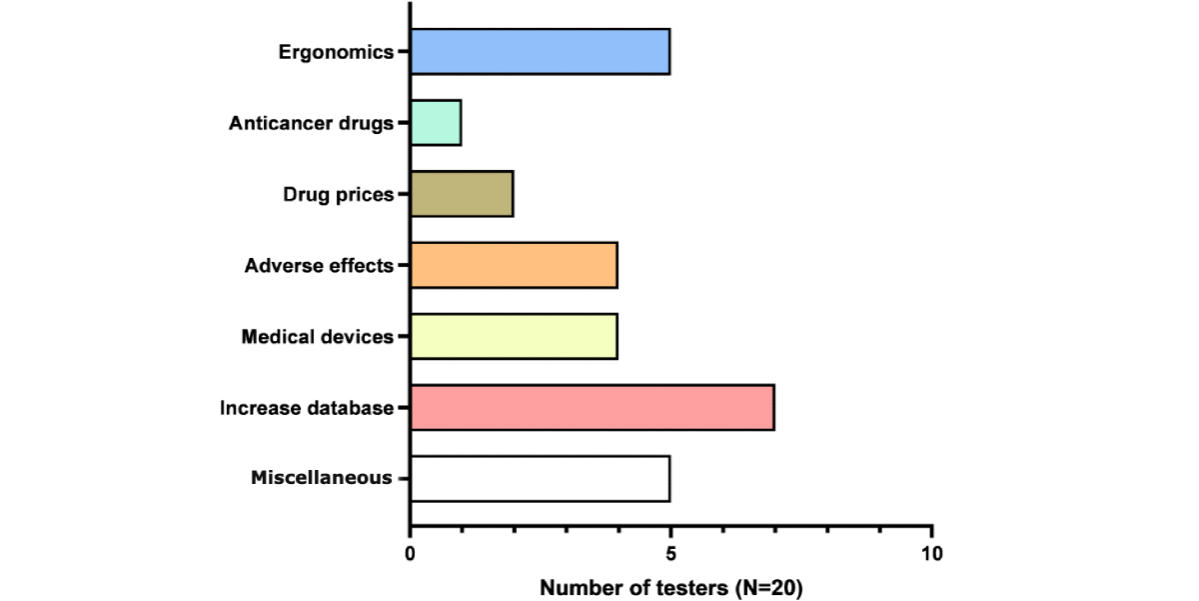
Suggestions for future improvements (by topic) from testers after the trial session. Percentages were calculated on the basis of the 20 people who took part.

At the end of the beta tests, the main improvements that were suggested by the testers were related to the ergonomics (n=5, 25% of testers), as well as the implementation of the database (n=7, 35% of testers), with the inclusion of answers related to medical devices (n=4, 20% of testers), adverse drug effects (n=4, 20% of testers), and drug prices (n=2, 10% of testers), as shown in Figure 5.

## Discussion

### Principal Findings

To our knowledge, the chatbot we designed is the first to specifically target the hospital health staff and answer questions related to drug circuits and pharmacy organization. There are currently very few chatbot-type solutions specifically dedicated to the hospital setting, and almost all of the existing literature deals with chatbots in relation to patients [15,16]. The perception of the chatbot tool by physicians has already been studied [17]. In this case, the chatbot made it possible to provide prescription aid but also information on drugs, such as our chatbot. In particular, the authors concluded that the chatbot would perfectly fit in the daily medical practice and that it was positively perceived by the physicians. However, the main limitation of the work remained the lack of information included in the database.

During the trial session, the beta testers found our chatbot to be fast, user-friendly, and aesthetically pleasing. The overall satisfaction as well as the actual performance of the chatbot were considered good (all scores were higher than 7 out of 10), and all testers explained that they would want to continue using the tool if its database is widened. They also declared that the chatbot could help them during the COVID-19 crisis to obtain reliable information they need about vaccination and easily respond to patient requests. The database was thus improved to take into account the suggestions of testers, and in doing so, expand the area of competence of the chatbot. We, therefore, added topics regarding drug prices, adverse drugs effects, and some questions dealing with anticancer drugs. We also added recent data regarding the management of vaccination of pregnant women, in accordance with the French Reference Center on Teratogens recommendations.

Thus, the chatbot solution developed and implemented in our hospital appears to be a useful and reliable tool to address the common drug- and pharmacy-related questions encountered by nurses and other hospital caregivers at any time during their practice. It is accessible, implemented in the hospital intranet that health professionals are accustomed to using, and easy to use.

### Limitations

We attempted to include as much data as possible in the chatbot system to answer caregivers’ questions, taking into account the limited resources (both human and technical) allocated to its development. The small number of beta testers (n=20) is, therefore, a limitation of our work, and there are also other data that need to be integrated into the chatbot. One of the most frequent requests concerned drugs’ stability. To provide an answer, we included cross-tables that are concise, extremely dense, and not very user-friendly. Another solution would be to redirect the user to a reliable website such as specialized websites, but this would force users to transcribe their information and use another tool, which is highly likely to lead to a loss of support toward the chatbot. Finally, the topic of medical devices is also under study; however, the main difficulty remains the complex nomenclature and the extremely variable names that a single device may have. This topic has not been included in the chatbot’s scope, and currently, the chatbot simply invites users asking a question about it to contact specifically pharmacists or pharmacy assistants working in this field.

Another technical limitation of the chatbot tool is the fact that it was not possible to interface the chatbot with our business software because of an incompatible application programming interface. This limitation is likely to evolve in the future, with software companies taking into account the need for flows’ interoperability.

When interpreting the results, it is also important to note that the relevance score evaluated during beta tests might be overestimated because of interactions that were interrupted by testers before the end of the study period. These aborted interactions are not recorded in the performance ratio included in the back office.

On the user’s side, the degree of mastery of computer tools generally determines the handling and the acceptance of the chatbot. There is also resistance to change, as the use of the chatbot is not currently part of the habits and routine of the staff. It is, therefore, necessary to facilitate its use as much as possible, and its relevance should be demonstrated in the early stages of use to promote its integration into the caregivers’ practice. Caregivers will include the chatbot in their practice in the long run only if its effectiveness and time-saving potential during searches for reliable information are clearly demonstrated. Should it fail to demonstrate these abilities, the deceptive effect is likely to prevail, and beyond the “novelty effect,” caregivers may lose interest in the project.

### Perspectives

Regular updating of the information integrated in the database is a central issue in maintaining a high level of reliability. This requires the appointment of a person who would be directly responsible for managing the chatbot. Thus, it is possible to entrust this task to a pharmacy intern, who could weekly consult the unanswered questions and integrate the corresponding information into the chatbot. We expect to implement these updates on a monthly to biannual basis according to the frequency of modification of the information and their levels of criticality. Moreover, the chatbot manager could also inform users in real time about significant breaks or important news, using the “programmed events” feature. It consists of a banner—that would appear when the chatbot is launched—to feature important information. However, this would require close collaboration with the procurement sector of the hospital pharmacy.

In the future, it may also be possible to expand the scope of the chatbot’s skills even further, by including, for example, a list of medical devices available in the hospital. However, given the extremely large number of references, it will be necessary to implement a relatively effective indexing or search system to better guide the user among the complex diversity of labels. Another major element in the future of the chatbot is its deployment on the 2 hospital sites so that all hospital caregivers can use it. In order to do so, it is necessary to take into account the specificities of each site; for example, drug availability. In another update, we could also include a list of verified, up-to-date websites to convert this tool to a weapon against medical misinformation. In the context of health crises, the chatbot may be a relevant means of information to transmit homogeneous information to all caregivers of our hospital. The development of different type of interfaces, such as smartphones or a speech-to-text option, may also be a relevant perspective to improve the ergonomics and adherence of caregivers.

Finally, the chatbot could become an effective training tool for pharmacy technicians. Indeed, the chatbot would allow them to answer a wide variety of drugs-related questions on their own and more easily, without necessarily having to ask a referent pharmacist. Of course, questions about the patient’s clinic or specific elements of drugs will always require pharmaceutical expertise, but we believe that the chatbot may allow pharmacists to optimize their time for value-added tasks. The tool could also constitute an essential element for the training of new staff and for personnel who may move between care units for organizational purposes.

### Conclusions

The chatbot seems to be a useful tool for hospital caregivers, helping them obtain the information they need about drugs and pharmacy organization. To our knowledge, this is the first time that such a tool has been designed for hospital caregivers. It is, therefore, crucial to continue developing it using tests with and implementing additional data to improve its performance. Several aspects still need to be improved, especially in relation to the scope of the chatbot’s skills. Eventually, the technology could be shared with other institutions, or even to websites, such as those of learned societies. The chatbot could also become an essential component for the training of new health care staff and on the occasion of staff changes in different departments for organizational purposes.

## Appendix

Multimedia Appendix 1 Schema of the chatbot's decision algorithm regarding the availability of a drug.

## Abbreviations

ADDIE: Analysis, Design, Development, Implementation, and Evaluation
WIOS: Weekday Inpatient Oncology Service
