# Supplementary figures and images for: Answering Hospital Caregivers’ Questions at Any Time: Proof-of-Concept Study of an Artificial Intelligence–Based Chatbot in a French Hospital
Source: JMIR Hum Factors. 2022 Oct 11;9(4):e39102. doi: 10.2196/39102 (PMC9555819; doi:10.2196/39102)

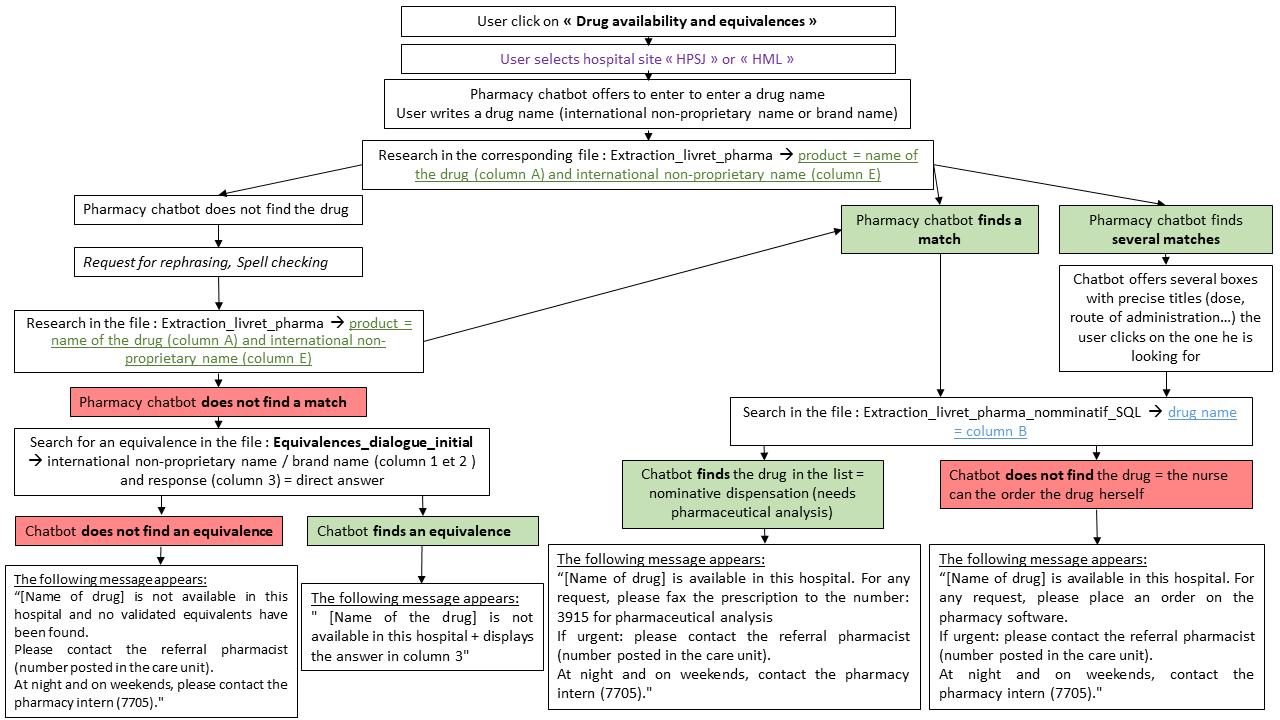

Supplement: Multimedia Appendix 1 [file humanfactors_v9i4e39102_app1.png]
